# Ossification of the Choroid

**Published:** 1900-03

**Authors:** James Moores Ball

**Affiliations:** St. Louis; President of the St. Louis Academy of Medical and Surgical Sciences; Professor of Ophthalmology in the St. Louis College of Physicians and Surgeons; Oculist to the City Hospital, the Jefferson Hospital, St. Joseph’s Sanatorium, and the St. Louis and San Francisco Railway


For Texas Medical Journal.

### Ossification of the Choroid.

BY JAMES MOORES BALL, M. D., ST. LOUIS.
President of the St. Louis Academy of Medical and Surgical Sciences; Professor of Ophthalmology in the St. Louis College of Physicians and Surgeons; Oculist to the City Hospital, the Jefferson Hospital, St. Joseph's Sanatorium, and the St. Louis and San Francisco Railway.

Ossification of the choroid is a rare condition, which I have met with three times in my practice. As an example of the conditions leading to a deposit of bone in this part of the eye I will cite a recent case:

Mr. W. R. O., aged 36, was sent to me by Dr. G. W. Vernon, of Dexter, Mo., on February 16, 1900. Thirty-three years ago the patient was struck in the right eye by a piece of gun-cap. The eye became blind, but did not cause trouble until six weeks ago, when it became painful. His family physician recognized the danger of the patient, and sent him to me. I found the eye blind and exceedingly tender to the touch. The slightest pressure would cause the patient to flinch. The good eye was not irritated; did not water on exposure to light and presented a normal appearance. The patient states that whenever he reads the blind eye pains him. An enucleation was advised, and I made the operation at once. After removal it was found that the eye was abnormally hard. I remarked to the class that this was an example of ossification of the choroid. This opinion was confirmed by pathologic examination. The interesting features of this case are the long duration of the cyclitic process without the production of sympathetic ophthalmia, and the reference of pain to the blind eye on accommodative effort.

### PATHOLOGY.

The formation of bone in the eyeball occurs only in the choroid. Although the choroid and iris have practically the same structure, no case has yet been recorded of bone formation in the iris. Ossification of the choroid occurs in old cyclitic eyes. The bony tissue may be in the form of plates or of a shell occupying the posterior one-third or one-half of the globe. The mass of bone lies on the inner surface of the choroid. The outer surface is smooth, the inner ragged. The color of the mass is brownish. Sometimes the bony formation is in the shape of a ring located around the optic disc or on the inner surface of the ciliary body. To form a shell in the choroid is said to require a period of fifteen years or more; the small plates or spiculæ can form in three years. Microscopic section shows true bone cells with lacunæ and canaliculi.

### SYMPTOMS.

Eyes containing bone do not present symptoms differing from those found in old cases of cyclitis, as a rule. Sometimes the presence of ossification of the choroid can be determined before enucleaation, but generally it is not suspected until the operation is well under way. Any old cyclitic eye which is tender on pressure should be removed, to preserve the integrity of the other eye.

